# Exogenous dopamine reduces GABA receptor availability in the human brain

**DOI:** 10.1002/brb3.484

**Published:** 2016-05-05

**Authors:** Hans C. Lou, Astrid Rosenstand, David J. Brooks, Dirk Bender, Steen Jakobsen, Jakob U. Blicher, Kim V. Hansen, Arne Møller

**Affiliations:** ^1^ CFIN and Pet CenterAarhus UniversityNorregade 448000AarhusDenmark; ^2^ Dept. OphtalmologyRigshospitaletGlostrupCopenhagen UniversityDenmark

**Keywords:** Dopamine, GABA, insula, medial prefrontal/anterior cingulate, self‐awareness

## Abstract

**Background:**

While it has recently been shown that dopamine release stimulates conscious self‐monitoring through the generation of gamma oscillations in medial prefrontal/anterior cingulate cortex, and that the GABAergic system is effective in producing such oscillations, interaction of the two transmitter systems has not been demonstrated in humans. We here hypothesize that dopamine challenge stimulates the GABA system directly in the medial prefrontal/anterior cingulate region in the human brain.

**Methods:**

Positron emission tomography (PET) with the GABA receptor *α*1/*α*5 subtype ligand [^11^C] Ro15‐4513 was used to detect changes in GABA receptor availability after clinical oral doses of levodopa in a double blind controlled study.

**Results:**

We here provide the first direct evidence for such coupling in the cerebral cortex, in particular in the medial prefrontal anterior cingulate region, by showing that exogenous dopamine decreases [^11^C] Ro15‐4513 binding widely in the human brain compatible with a fall in *α*1 subtype availability in GABA complexes due to increased GABA activity.

## Introduction

Cognition depends on paralimbic medial prefrontal/anterior cingulate and medial parietal hubs (Lou et al. 2004; Luber et al. 2012) and their rich network of highly oxygen‐demanding, fast‐spiking GABAergic interneurons (Tomasi et al. 2013). The network is regulated by dopamine, which in humans stimulates medial prefrontal/anterior cingulate cortex and insula, thereby generating gamma oscillations (Joensson et al. 2015) essential for cognitive function (Voss et al. 2014). The high oxygen requirement of these hubs (Tomasi et al. 2013) makes the system vulnerable in penuria, and dysfunction is associated with disorders like ADHD (Castellanos et al. 2008), autistic spectrum disorder (Assaf et al. 2010), schizophrenia (Gallinat et al. 2004), addiction (Romer et al. 2013), and dementia (Liu et al. 2014), and can follow traumatic brain injury (Ham et al. 2014). It is, therefore, important to determine how the information gathered in animal models on the molecular arrangement of dopamine/GABA interaction (Sesack et al. 2003) translates into the human brain. This is particularly the case for the medial prefrontal/anterior cingulate region which facilitates attention and self‐awareness. With that aim we here examined the effect of oral l‐dopa, a dopamine prodrug, on the activation of the GABA system in the human living cerebral cortex. The idea that dopamine release regulates cognition, including conscious experience, via the GABAergic system is based on the finding that dopamine increases confidence and accuracy of seeing rapidly presented words (Lou et al. 2011), and on the fact that GABAergic interneurons are instrumental in mediating cognition in rodents (Sohal et al. 2009). The hypothesis is further strengthened by the recent demonstration that dopamine release in the medial prefrontal/anterior cingulate cortex activates self‐monitoring and metacognition (Joensson et al. 2015). Theoretical aspects of interaction between dopamine and GABA function have been studied in detail in a recent neurocomputational model by Lew and Tseng (2014).

Direct experimental approaches have been scarce, but recently it has been demonstrated that extracellular striatal dopamine elicits opposing effects on the GABA system in rodents: dopamine release *activates* synaptically located GABA_A_ receptor subtypes, including alpha 5, but *inhibits* tonic GABA_A_ currents via extrasynaptically located tonic level GABA_A_ receptors (Hoerbelt et al. 2015). These effects of dopamine mimic similar opposing effects of extracellular GABA (Ransom et al. 2013), and are in accordance with the concept that tonic GABA currents are a homeostatic regulator responding to localized synaptic stimulation of GABA receptors by decreasing excitability in the network (Stokes et al. 2014). Here, we provide the first direct in vivo evidence for such interaction in humans by showing that an exogenous dopamine challenge in physiological relevant dosage decreases GABA receptor availability, reflecting increased GABA activity.

## Participants and Procedures

The study was approved by the local ethical board (Region Midtjylland). After information given orally and in writing, and consent, nine healthy males were studied. This provides 80% power to detect a 20% reduction of anterior cingulate Ro 15‐4513 binding at *P* = 0.05. The participants were without current medication. None had a history of mental or neurological disorders, or a history of addiction, with the exception that two had been smokers 10 and 15 years ago, respectively. Median age was 29 years, range 24–47. The PET ligand [^11^C]Ro15‐4513 is sensitive to interstitial GABA levels evidenced as a fall in availability of *α*1 subtype sites on the GABA complex for its binding (Semyano et al. 2004; Stokes et al. 2014). While [^11^C]Ro15‐4513 binds to both the *α*1 and *α*5 receptor subtypes, the *α*1 subtype is present at a greater density and increases in brain GABA have been shown to reduce its availability, while *α*5 binding site availability is either unaffected or mildly elevated. In the present study, it was not possible to separate signals from *α*1 and *α*5 subunits of the GABA complex as we did not have an arterial input curve so we have assumed that any decreased Ro 15‐4513 binding seen represented competitive occupancy of *α*1 sites by endogenous GABA (Lingford‐Hughes et al. 2002).

Each participant was studied twice in counterbalanced order with an interval of at least several days. We used PET following an oral l‐dopa/dopamine challenge or placebo in identical capsules given orally containing either sinemet (MSD, 100 mg l‐dopa plus 25 mg carbidopa) or placebo (starch). The medication was unknown to the participants and experimenters responsible for PET injection and calculation of binding potentials. The administration of l‐dopa or placebo was done between 30 and 45 min prior to intravenous injection of [^11^C]Ro15‐4513. This interval is based on the interval for obtaining peak plasma concentrations and clinical effects on parkinsonism after oral sinemet medication (Joensson et al. 2015).

## Results and Discussion

**Figure 1:**
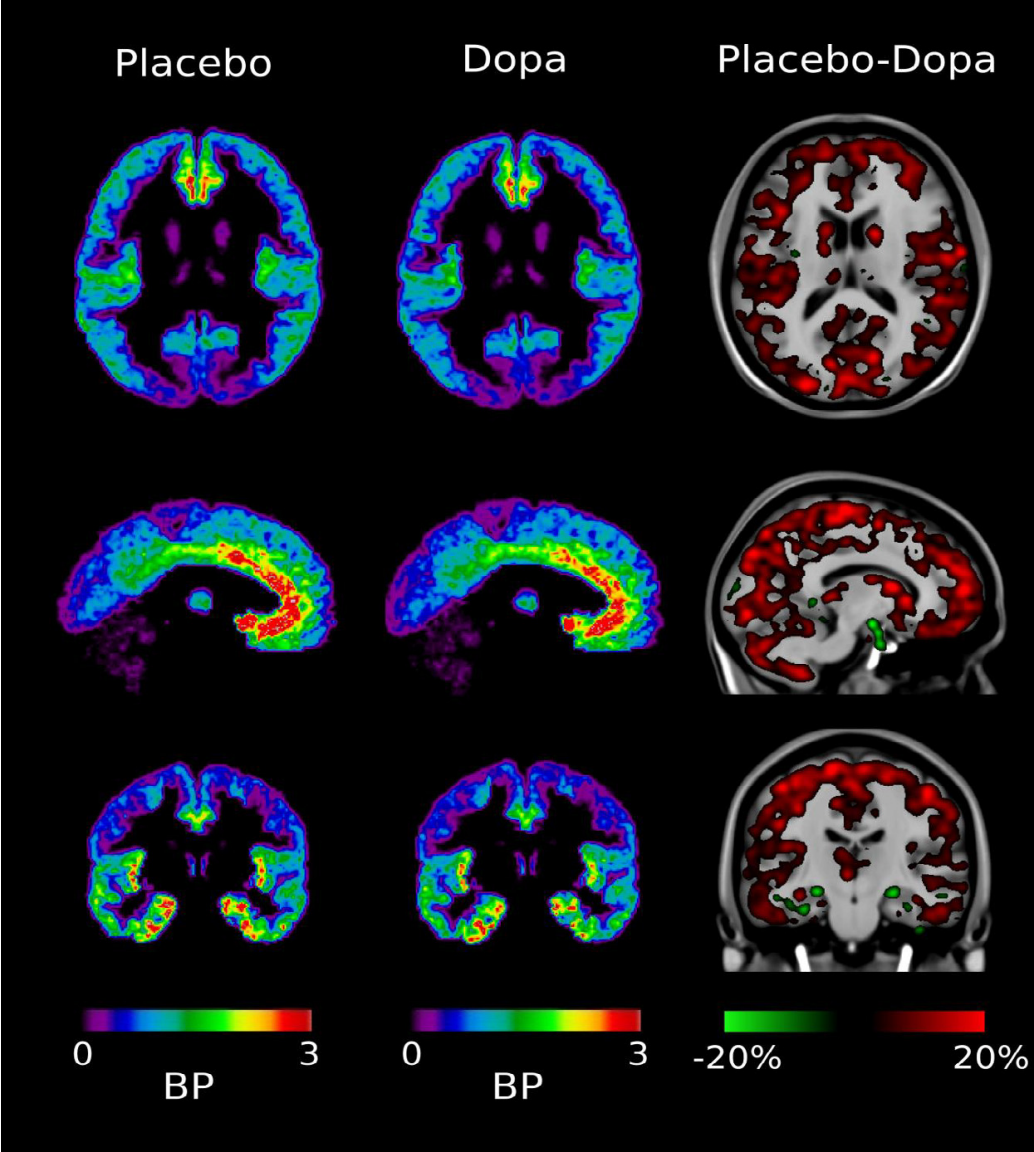
Distribution of [^11^C]Ro15‐4513. After placebo, the ligand was bound primarily in medial prefrontal/anterior cingulate cortex and left and right insula. After dopamine challenge ligand binding is reduced. This is seen more clearly by subtraction, with a general reduction of between 5% and 20% throughout gray matter (BP 100x [placebo minus dopamine]/placebo). In the hippocampal regions there were small foci of increased BP.

The distribution of [^11^C]Ro15‐4513 binding with placebo is shown in Figure 1. After placebo administration, the ligand is bound primarily in medial prefrontal/anterior cingulate cortex and left and right insula. The cerebellum provides a reference for nonspecific tissue binding of the tracer. After l‐dopa, [^11^C]Ro15‐4513 binding is reduced, which can be seen more clearly by subtraction. There is a reduction of between 5% and 20% throughout most of gray matter regions (BP 100x [placebo minus dopamine]/placebo). Small foci of BP *increases* were, however, present in the hippocampi, possibly reflecting the high concentration of D4 dopamine receptors there which inhibit interneuron GABA release when activated (Romo‐Parra et al. 2005).

**Figure 2:**
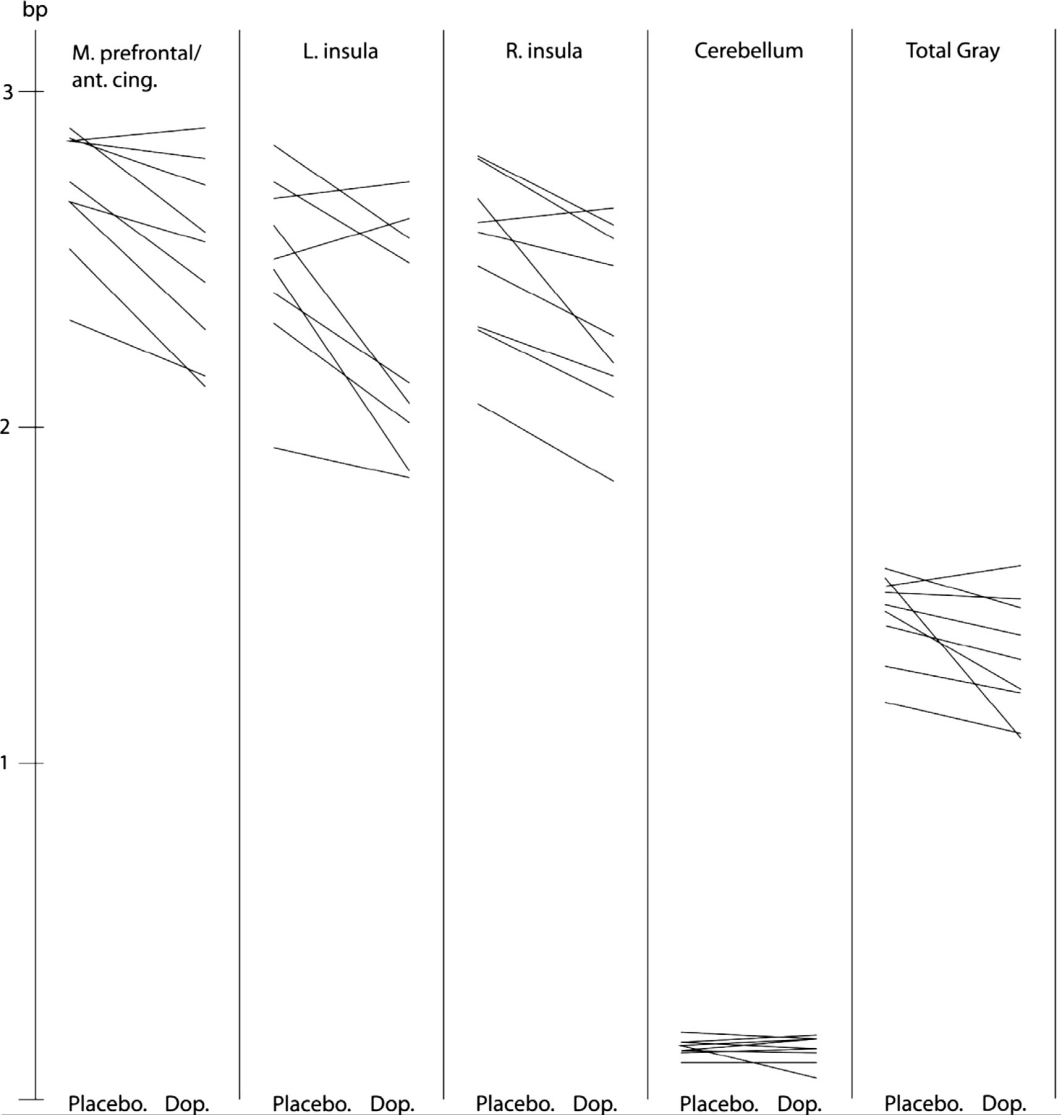
Quantification in medial prefrontal/anterior cingulate region, left and right insula, cerebellum, and whole gray matter. Using parametric modeling, a decrease in [^11^C]Ro15‐4513 BP is apparent overall after l‐dopa, except in cerebellum. For statistics, see text.

Quantification using parametric modeling is shown in Figure 2 for medial prefrontal/anterior cingulate region, left and right insula, cerebellum, and whole gray matter. For a composite gray matter region, a one‐sample *t*‐test showed that l‐dopa was associated with an 11% mean decrease in [^11^C]Ro15‐4513 BP (*t* = −2.29, 9 degrees of freedom, *P* = 0.048). There was a highly significant effect of regions (linear mixed model, *P* = 0.000049). Because dopamine has previously been shown to stimulate electromagnetic activity and self‐awareness mainly in the medial prefrontal/anterior cingulate and insula regions (Joensson et al. 2015), we a priori calculated BP changes in these regions separately and compared these with cerebellum and whole gray matter, using two‐tailed *t* tests with Bonferroni corrections and a cut off at *P* = 0.01. The BP of the GABA ligand decreased significantly with l‐dopa in the medial prefrontal/anterior cingulate and right insula regions (by 18%, *t* = −3.63 *P* = 0.0054, and 18%, *t* = 3.85 *P* = 0039, respectively), with no significant effect in the other regions.

Thus, the main findings were (1) preferential binding of [^11^C]Ro15‐4513 to *α*1/*α*5 subunits on GABA complexes in the medial prefrontal/anterior cingulate region and (2) a widespread and proportional dopamine‐induced decrease in binding potentials for the GABA ligand [^11^C]Ro15‐4513 (Lingford‐Hughes et al. 2002).

The present results account for the effect of dopamine in its regulation of self‐awareness via direct action on the GABA system in the medial prefrontal/anterior cingulate cortex. Similarly, the direct effect of dopamine on GABA in right insula, rather than the left, may explain the preferential role of right hemisphere in self‐recognition (Keenan et al. 2001). The close cortical interaction of the two transmitter systems broadens the scope for pharmacological intervention in the many human disorders where the medial prefrontal region is dysfunctional. It may open new perspectives for treatment.

## Methods

### Magnetic resonance imaging

All participants were scanned with a 3T MR system pre‐registration with PET (Siemens Trio, Erlangen, Germany). A T1 MPRAGE scan (TR/TE 2420/3.7 msec, 1 mm isotropic resolution, scan time 5½ min) was performed.

### Synthesis and purity of [^11^C]15‐4513

[^11^C]15‐4513 was synthesized by the adoption of the procedure of Halldin et al. (1992). Briefly, ^11^CO_2_, either produced with the PET centers GE PETtrace or IBA18/18 cyclotron, was converted to [^11^C]methyl iodide applying a GE Healthcare MeI Microlab. [^11^C]methyl iodide was trapped at room temperature in a solution of 0.3–0.5 mg Ro44‐3902 (ABX advanced biochemical compounds, Radeberg, Germany) in 0.3 mL DMF, containing 0.5–1 mg NaH (60% dispersion in mineral oil; Sigma‐Aldrich A/S, Brøndby, Denmark). After heating for 1 min at 50°C [^11^C]15‐4513 was isolated by preparative HPLC applying a 250 × 10 mm Phenomenex Luna C18(2) column and a mixture of 70 mmol/L NaH_2_PO_4_ buffer and acetonitrile (70:30). The fraction containing [^11^C]15‐4513 was collected, diluted with 50 mL of sterile water. Afterward [^11^C]15‐4513 was trapped on Water Sep‐Pak A/S, Hedehusende, Denmark^®^ C‐18 Plus light cartridge. Remaining acetonitrile was removed by flushing the Sep‐Pak cartridge with 20 mL of sterile water. [^11^C]15‐4513 was desorbed with 1 mL of sterile ethanol followed by 9 mL of saline. The final solution was sterile filtered via 0.22‐*μ*m sterile filter into a sterile product vial. This procedure yielded 1–6 GBq of [^11^C]15‐4513 within 35 min and radiochemical purities >95%. Amounts of nonradioactive [^12^C]15‐4513 were in the range 0.3–3.5 *μ*g/mL (1–11 nmol/mL). Interestingly, we found increased radiolysis on the C‐18 Sep‐Pak affecting the radiochemical purity at very high radioactivities.

### Positron emission tomography

Participants were placed in the scanner with the orbitomeatal line parallel to the transaxial plane of the tomograph. Head position was monitored via laser crosshairs and video camera. In order to correct for attenuation of emitted radiation by skull and tissues, a transmission scan was acquired using a single rotating photon point source of 150 MBq of ^137^Cs. Participants were pretreated with placebo or 100 mg l‐dopa + 25 mg carbidopa given orally to block peripheral metabolism of l‐dopa, thereby increasing tracer delivery to the brain. This was done 30–45 min prior to injection of 400 (range 375–425) MBq [^11^C]Ro15‐4513 through a cubital vein in 10 mL of normal saline over 30 sec. Three‐dimensional PET was recorded over 60 min, using an ECAT EXACT HR++ (CTI/Siemens 966; Siemens PET/CT Biograph, Erlangen, Germany) camera, which covers an axial field of view of 23.4 cm and provides 95 transaxial planes. The tomograph has a spatial resolution of 4.8 + 0.2 mm FWHM (transaxial, 1 cm off axis) and 5.6 mm + 0.5 mm (axial, on axis) after image reconstruction. PET and MRI were coregistered to a common MR T1 atlas from Montreal Neurological Institute (MNI) (Collins et al. 1998) using PMOD (PMOD Technologies Ltd., Zurich, Switzerland). The kinetic parameters of [^11^C]Ro15‐4513 were determined by the SRTM2 method using cerebellum as reference (Wu and Carson 2002). No FDR or cluster corrections were used as the regions were predefined.

## Conflict of Interest

The authors declare no competing financial interest.
